# Radiation Damage in Biomolecules and Cells 2.0

**DOI:** 10.3390/ijms24043238

**Published:** 2023-02-06

**Authors:** Mario P. Carante, Ricardo L. Ramos, Francesca Ballarini

**Affiliations:** 1 Physics Department, University of Pavia, Via Bassi 6, 27100 Pavia, Italy; 2 INFN (Italian National Institute for Nuclear Physics), Sezione di Pavia, Via Bassi 6, 27100 Pavia, Italy

It is well known that ionizing radiation, when it hits living cells, causes a plethora of different damage types at different levels. This begins with damage to DNA and other biomolecules and extends to damage involving tissues, organs, and even the whole organism.

Although radiobiological damage may occur via the so-called “bystander effects”, not involving DNA [1], the double helix is widely considered as the most important target. The initial DNA damage, produced either by direct energy deposition in the DNA atoms (“direct damage”) or following interaction with free radicals (“indirect damage”), is then recognized and processed by specific enzymes. In the case of incorrect DNA repair, the initial damage can evolve within a few hours into chromosomal aberrations, which consist of large-scale rearrangements of chromatin fragments [2]. In turn, some chromosome aberrations (typically dicentrics, rings, and excess acentric fragments) can lead to cell death [3], while others (such as some reciprocal translocations) can cause cell conversion to malignancy and even cancer [4].

All of these processes depend on a variety of factors, including the dose of radiation, the dose-rate, and the radiation quality (i.e., particle type and energy). Target cell characteristics, including the cell radiosensitivity and its chromatin organization in the cell nucleus, are also involved [5,6]. In particular, the RBE (relative biological effectiveness), defined as the ratio between the photon dose and the ion dose necessary to obtain the same biological effect, is widely used in radiobiology. It enables comparison of the biological effectiveness of X- or gamma-rays with that of protons, He-ions, and heavier ions. These include C-ions, which are used in cancer therapy, and Fe-ions, which are present in space radiation and are particularly effective at producing a high number of small DNA fragments [7] and chromosome aberrations [8].

It is, therefore, mandatory for the scientific community to continuously update and improve the knowledge regarding the mechanisms governing the induction of radiation effects in biological targets, as well as to apply the acquired information in order to optimize the use of ionizing radiation and the corresponding radiation protection strategies.

In this framework, this Special Issue reports experimental and theoretical works on the effects of ionizing radiation at the DNA level, as well as possible applications in cancer therapy and space radiation protection. More specifically, the work by Faretta and co-workers [9] deals with the role played by 53BP1, with respect to DNA damage response (DDR) and p53 action. Since the localization of the 53BP1 protein represents the key to deciphering the network of activities exerted in response to stress, the authors present an automated microscopy strategy for an image cytometry protocol to analyze the evolution of DDR, as well as to demonstrate how 53BP1 moves from damaged sites to the nucleoplasm, interacting with p53 and enhancing its transcriptional regulation. Molecular interactions have been quantitatively described and spatio-temporally localized at the highest spatial resolution by a simultaneous analysis of the cell-cycle progression impairment. The high statistical sampling of the protocol allowed a detailed quantitative description of the molecular events following DSBs (double-strand breaks) formation to be provided. Finally, single-molecule localization microscopy (SMLM) analysis confirmed the p53–53BP1 interaction at the tens of nanometers scale during the various response phases.

The paper by Zhu et al. [10] presents a theoretical study simulating DNA response after proton irradiation. A chromatin fiber model, together with a new physics constructor with the ELastic Scattering of Electrons and Positrons by neutral Atoms (ELSEPA) model, was applied to describe the DNA geometry and the physical stage of water radiolysis by means of the Geant4-DNA toolkit, respectively. Three key parameters (energy threshold model for strand breaks, physics model, and maximum distance to distinguish clusters of double-strand breaks) for DNA damage scoring were analyzed to investigate their impact on the uncertainties affecting DNA damage. Following comparison with the experimental data and published results, the authors predicted the yield of different DNA damage types. The results showed that the difference in the physics constructor can cause up to 56.4% in the DSB yields. The latter were quite sensitive to the energy threshold for strand breaks and the maximum distance to classify the DSB clusters, which were more than 100 times and 4 times higher with respect to the default configurations, respectively.

The Geant4-DNA toolkit was also exploited in the work by Thibaut et al. [11]. They presented a new geometrical model of an endothelial cell nucleus and DNA distribution according to the isochore theory, where heterochromatin and euchromatin compaction are distributed along the genome based on five different families (L1, L2, H1, H2, and H3). Each family is associated with a different hetero/euchromatin rate, related to its compaction level. To compare the results with those obtained using a previous method of nuclear geometry, simulations were performed for protons with linear energy transfer (LET) values of 4.29 keV/µm, 19.51 keV/µm, and 43.25 keV/µm. The organization of the chromatin fiber at different compaction levels linked to isochore families increased the DSB yield by 6–10%, and allowed the most affected part of the genome to be identified. These new results indicate that the genome core is more radiosensitive than the genome desert, with a 3–8% increase in damage depending on LET. More generally, this work highlighted the importance of using realistic distributions of chromatin compaction levels to calculate radiation-induced damage by means of Monte Carlo simulations.

Mairani and co-workers [12] investigated the importance of DNA damage and repair processes for the induction of cell death, as well as for the accurate evaluation of the RBE, to support ion-beam cancer therapy. The approach is based on the “UNIfied and VERSatile bio response Engine” (UNIVERSE), a modeling framework developed to describe the effects of ionizing radiation on biological systems while considering numerous factors. Therein, the authors extended the UNIVERSE model to consider the time-dependent repair of isolated and complex DNA damage, and the resulting effects of the dose-rate. This development allowed trends to be predicted which were in line with measurements and models available in the literature, including an increased role of the dose-rate effect with an increasing dose and the saturation of the effect at high dose-rates. This approach, benchmarked against in vivo RBE data regarding the tolerance of the rat spinal cord, suggests the importance of considering dose-rate effects in clinical practice.

The work by Embriaco et al. [13] aimed to develop an approach to predict healthy tissue damage following ion irradiation, and to optimize hadrontherapy cancer treatments. The BIANCA (BIophysical ANalysis of Cell death and chromosome Aberrations) model, previously applied to predict the effectiveness of ion beams at killing tumor cells [14,15,16,17], was used to produce a radiobiological database describing the induction of chromosome aberrations in peripheral blood lymphocytes (PBL) as a function of ion type and energy. Since lymphocyte aberrations can be considered as indicators of hematologic toxicity, the database was used to calculate the corresponding RBE along therapeutic C-ion beams in order to evaluate the damage to healthy tissues. The differences found between the aberration RBE and the RBE for cell survival, which is routinely used for treatment plan optimization, suggests that calculating the beam biological effectiveness solely based on the cell survival RBE may lead to an underestimation of healthy tissue damage.

Finally, moving to the field of space radiation protection, the work by Cucinotta [18] presents a risk prediction model based on animal studies using high-LET radiation, denoted as the direct excess relative risk (DERR) model. The models currently used for risk estimation usually make extrapolations or estimations based on the biological effectiveness of gamma-rays, often ignoring the qualitative differences between low-LET radiation and high-LET radiation, which is typical of cosmic rays. This model, the parameters of which were fit to experimental data on carcinogenesis in mice exposed to heavy ions and fission neutrons, estimates the risk of cancer induction following exposure to cosmic rays. The author suggests that a significant reduction in uncertainty surrounding the results may be achieved by developing experimental models of the main tumors of which humans are at risk due to radiation.
